# Rare mandibular metastasis of neuroblastoma in a child: a case report

**DOI:** 10.3389/fdmed.2026.1885548

**Published:** 2026-08-27

**Authors:** Safaa El Omari, Malika Afif, Sana Bensouda, Samira El Arabi

**Affiliations:** Department of Pediatric Dentistry, Faculty of Dentistry, Hassan II University, Casablanca, Morocco

**Keywords:** case report, mandibular metastasis, neuroblastoma, oral lesion, pediatric oncology

## Abstract

Although neuroblastoma is a common malignant tumor in children and distant metastases are frequently observed, involvement of the mandible remains rare. We report the case of a 7-year-old boy presenting with a right mandibular mass associated with an oral lesion in the region of tooth 46, suggestive of a secondary lesion. Histopathological examination of the biopsy specimen confirmed the diagnosis, whereas imaging initially suggested lymphoma, soft tissue sarcoma, or osteosarcoma. Staging work-up revealed an adrenal neuroblastoma with multiple bone metastases. Given the extent of tumor involvement and the advanced stage of the disease, palliative chemotherapy was initiated. The clinical course was unfavorable, marked by the development of additional disseminated metastases, leading to the patient's death 14 months after the diagnosis of mandibular metastasis. This case highlights the importance of early multidisciplinary collaboration between pediatric dentistry and pediatric oncology teams to optimize diagnostic and therapeutic management in such patients.

## Introduction

Mandibular bone lesions in children are diagnostically challenging due to the broad range of possible etiologies and the considerable overlap in their radiological presentations. These lesions may arise from inflammatory or neoplastic processes, and can be either benign or malignant. In the pediatric population, primary mandibular tumors are uncommon, and most (70%–90%) are benign or of low malignant potential. In contrast, primary malignant tumors such as osteosarcoma or rhabdomyosarcoma remain exceptional (1). Even more rarely, children may present with maxillomandibular metastases, most often associated with malignancies such as neuroblastoma, Ewing sarcoma, Wilms tumor, retinoblastoma, or angiosarcoma (2–4).

Neuroblastoma is one of the most frequent extracranial solid tumors in childhood, accounting for approximately 8%–10% of pediatric malignancies. It arises from neural crest–derived cells, and its exact etiology remains unclear. According to the International Neuroblastoma Risk Group (INRG), it is the third most common solid tumor in children under 15 years of age, following leukemia and central nervous system tumors, and contributes to around 15% of pediatric cancer-related mortality (5). Its incidence varies across regions, with reported rates of 14.3% in Sweden, 7% in Europe, and 7.6% in Japan (6). In Morocco, it is ranked fourth among childhood cancers, after leukemia, lymphoma, and nephroblastoma (7).

At diagnosis, about 70% of neuroblastomas already present with metastatic disease. The most commonly affected sites include bone, bone marrow, lymph nodes, liver, and skin, while mandibular involvement remains uncommon. When present, maxillomandibular metastases generally reflect advanced systemic disease. Their recognition relies on careful clinical assessment, detailed medical history, and a high level of clinical suspicion when encountering atypical oral or maxillofacial lesions, which may allow earlier identification of an underlying malignancy (8, 9).

The aim of this case report is to highlight the crucial role of the dentist, particularly the pediatric dentist, in the early detection of oral manifestations that may reveal an underlying systemic malignancy. This is illustrated through a clinical case managed in the Pediatric Dentistry Department of the Casablanca Dental Consultation and Treatment Center (CCTD), Faculty of Dental Medicine, Casablanca, Morocco. The report also emphasizes the importance of close collaboration between dental and medical teams, which is essential to ensure early diagnosis and optimal multidisciplinary management.

## Case report

### Clinical case

A 7—year—old boy presented to the Pediatric Dentistry Department of the Dental Consultation and Treatment Centre (CCTD) in Casablanca with a marked, painful swelling involving the right side of the face.

The medical history revealed a neuroblastoma in remission for two years, previously presenting as an aggressive undifferentiated tumor of the right adrenal gland with mediastinal extension on imaging. Histopathological examination of the adrenal biopsy showed undifferentiated round cells with hyperchromatic nuclei arranged in sheets against a fibrillary background, consistent with an unfavorable histology undifferentiated neuroblastoma, further confirmed by positive immunohistochemical expression of CD56 and synaptophysin. Following induction chemotherapy, surgical resection demonstrated a maturing ganglioneuroma with 30% tumor necrosis, indicating a favorable therapeutic response.

The swelling had been progressively evolving over approximately three months, with no identifiable odontogenic cause.

**Figure 1:**
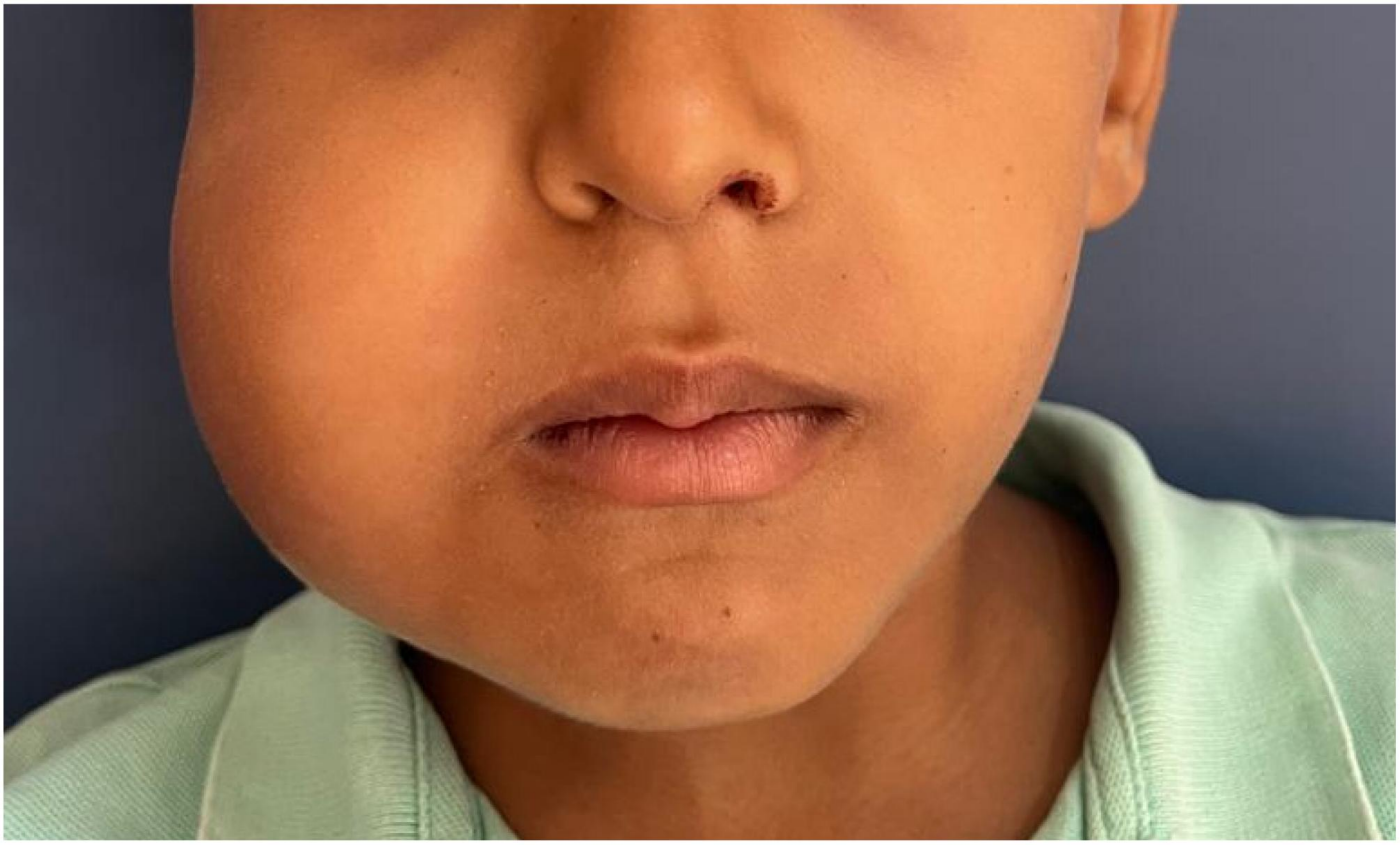
Facial asymmetry due to significant swelling of the right mandible.

Extraoral examination showed pronounced facial asymmetry secondary to a right mandibular swelling (Figure 1). Palpation revealed a hard, tender, unilateral cervical lymphadenopathy on the same side.

**Figure 2:**
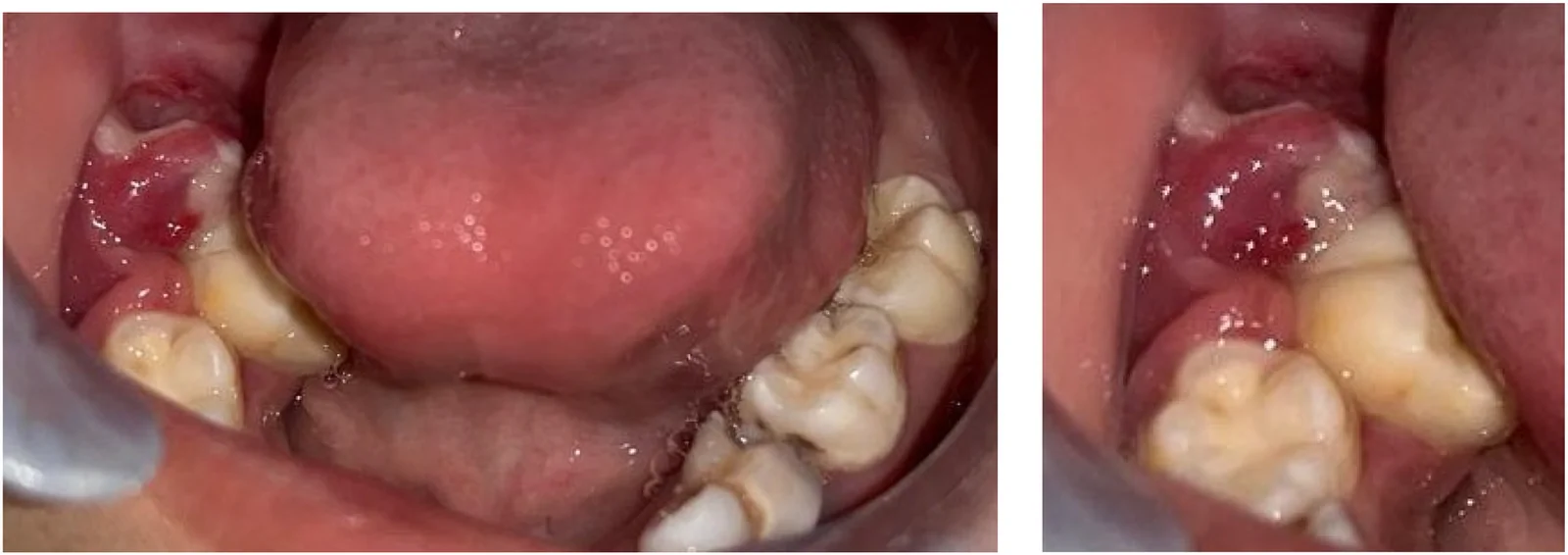
Intraoral photograph showing: hyperplastic gingival lesion around mobile tooth 46. Version of tooth 46 in lingual position. Whitish deposits in the retromolar region.

Intraoral examination revealed vestibular fullness in the right mandibular retromolar region, as well as a hyperplastic gingival lesion surrounding the mobile tooth 46, which was displaced lingually. Plaque-associated whitish deposits were also observed (Figure 2).

**Figure 3:**
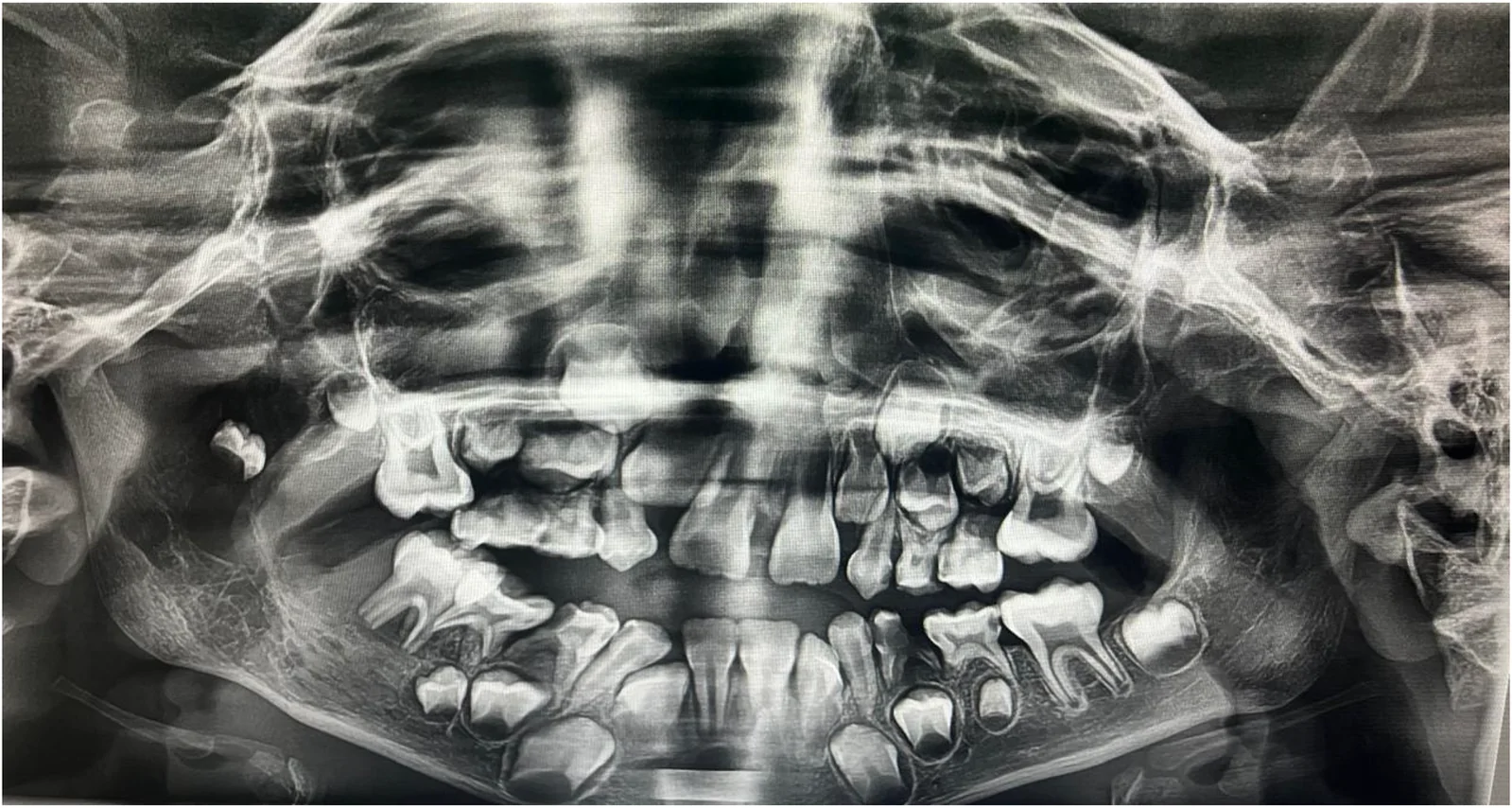
Panoramic radiograph showing: Non-circumscribed radiolucent image involving the body and ascending ramus of the mandible on the right side. 47: Delayed maturation and displacement of the germ in the axis of the ascending ramus on the right side. 46: Arrested root development and interradicular lysis. Tooth buds of 45 and 35: Size abnormality.

The panoramic radiograph revealed an extensive osteolytic lesion of the right mandibular ramus, with marked thinning and discontinuity of the inferior mandibular cortical border. A poorly defined radiolucent lesion was also observed in the right ascending ramus, suggestive of an infiltrative neoplastic process. At the level of tooth 46, extensive osteolytic changes were present, with arrest of root development and marked interradicular bone destruction. Displacement of the tooth germ of 47 was also noted, deviating from its normal position toward the axis of the mandibular ramus. In addition, the dental germs of teeth 35 and 45 appeared reduced in size, raising suspicion of a possible developmental morphological anomaly (Figure 3).

Further investigations were required to confirm the diagnosis and exclude other potential etiologies, including primary bone tumors, benign or pseudotumoral lesions, and metastatic involvement from another malignancy, particularly hematological disease.

**Figure 4:**
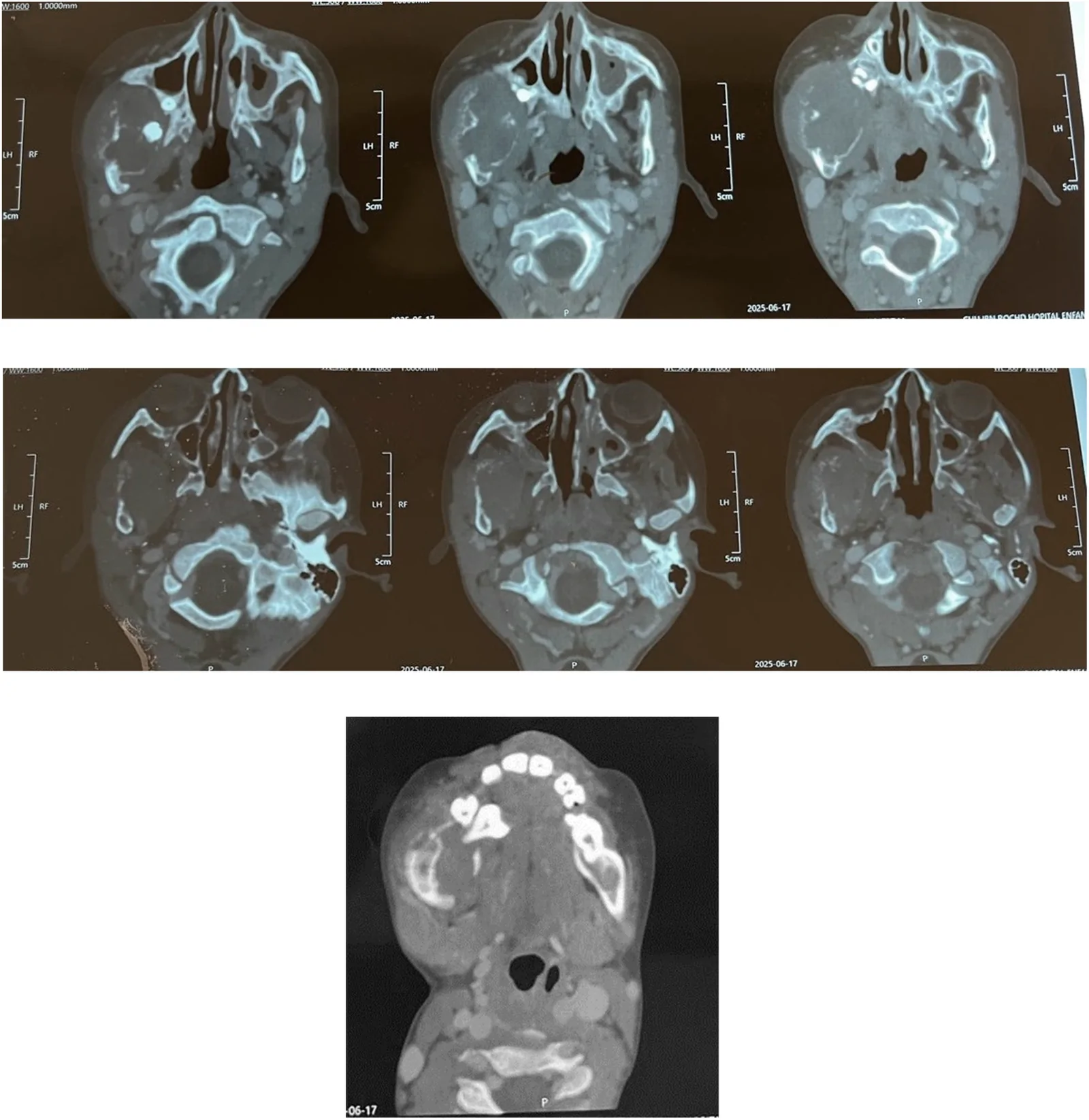
Sequential axial CT scans of the facial skeleton showing: An extensive multilocular osteolytic lesion of the right mandibular body and ramus. Marked cortical bone destruction with soft tissue extension. A lesion measuring approximately 4–5 cm in transverse diameter and 3–4 cm in anteroposterior diameter. Lingual displacement of tooth 46. Significant facial asymmetry.

The patient was therefore referred to his hematology-oncology team at the 20 Août Hospital in Casablanca. A computed tomography (CT) scan was performed as a first-line imaging modality, revealing extensive osteolytic destruction of the right mandibular body and alveolar ridge, lingual displacement of tooth 46, and a large soft tissue mass centered on the right mandibular ramus (Figure 4).

To guide the diagnosis, a biopsy of the oral lesion was performed by the referring physician. Histopathological examination of the mandibular biopsy revealed diffuse infiltration of the fibro-osseous tissue by a malignant small round cell tumor. The neoplastic cells exhibited hyperchromatic nuclei, scant cytoplasm, and a high nuclear-to-cytoplasmic ratio, with focal areas of crush artifact. These morphological findings were compatible with metastatic neuroblastoma and correlated with the histopathological features of the patient's previously diagnosed undifferentiated adrenal neuroblastoma, thereby supporting the diagnosis of mandibular metastatic recurrence. Extension workup included thoraco-abdomino-pelvic CT scan, MIBG scintigraphy, and PET scan.

## 10-month post-treatment follow-up

**Figure 5:**
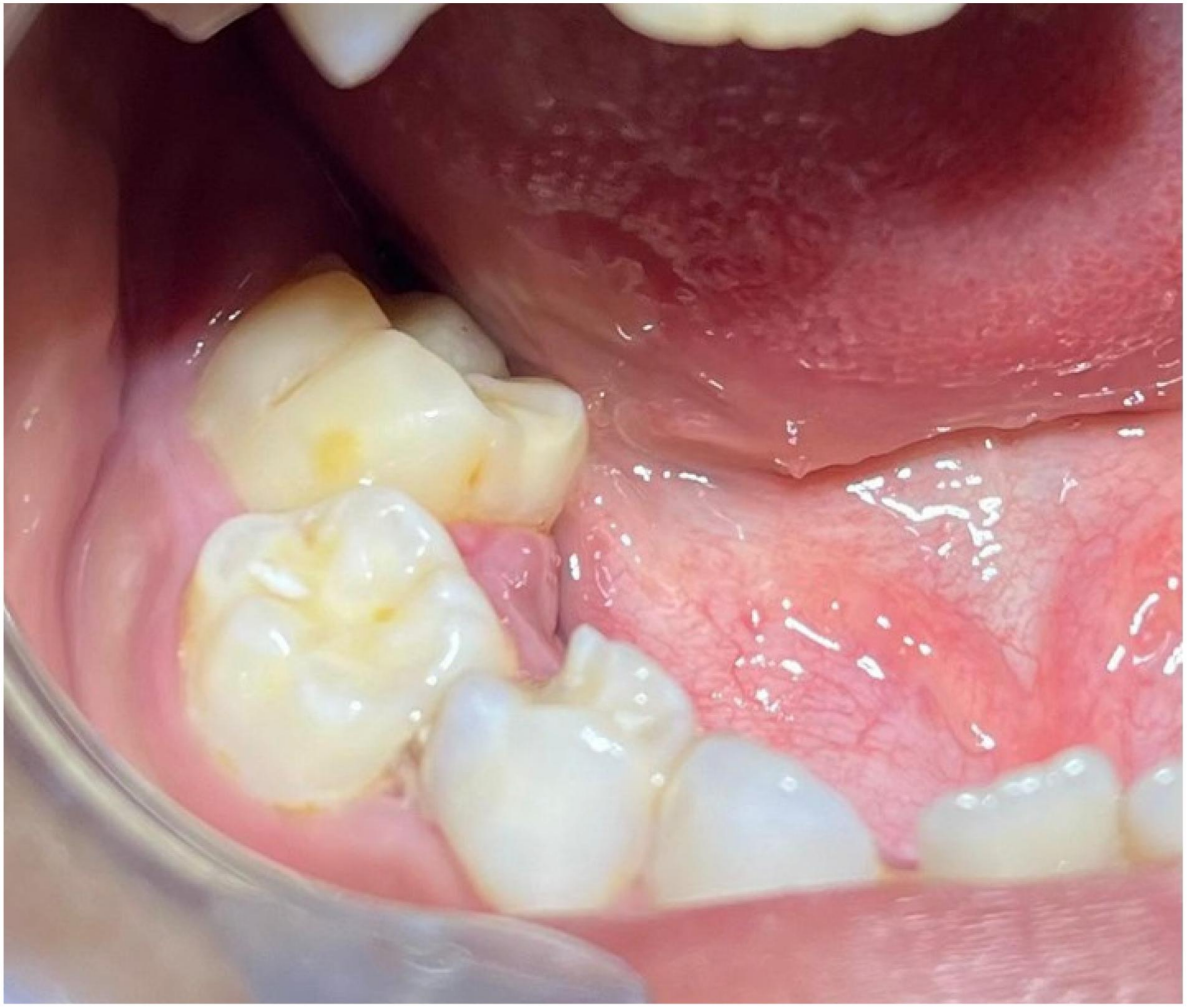
Intraoral photograph showing: marked regression of the hyperplastic lesion compared with the initial examination. Slight buccal repositioning of tooth 46 following the reduction in lesion volume.

**Figure 6:**
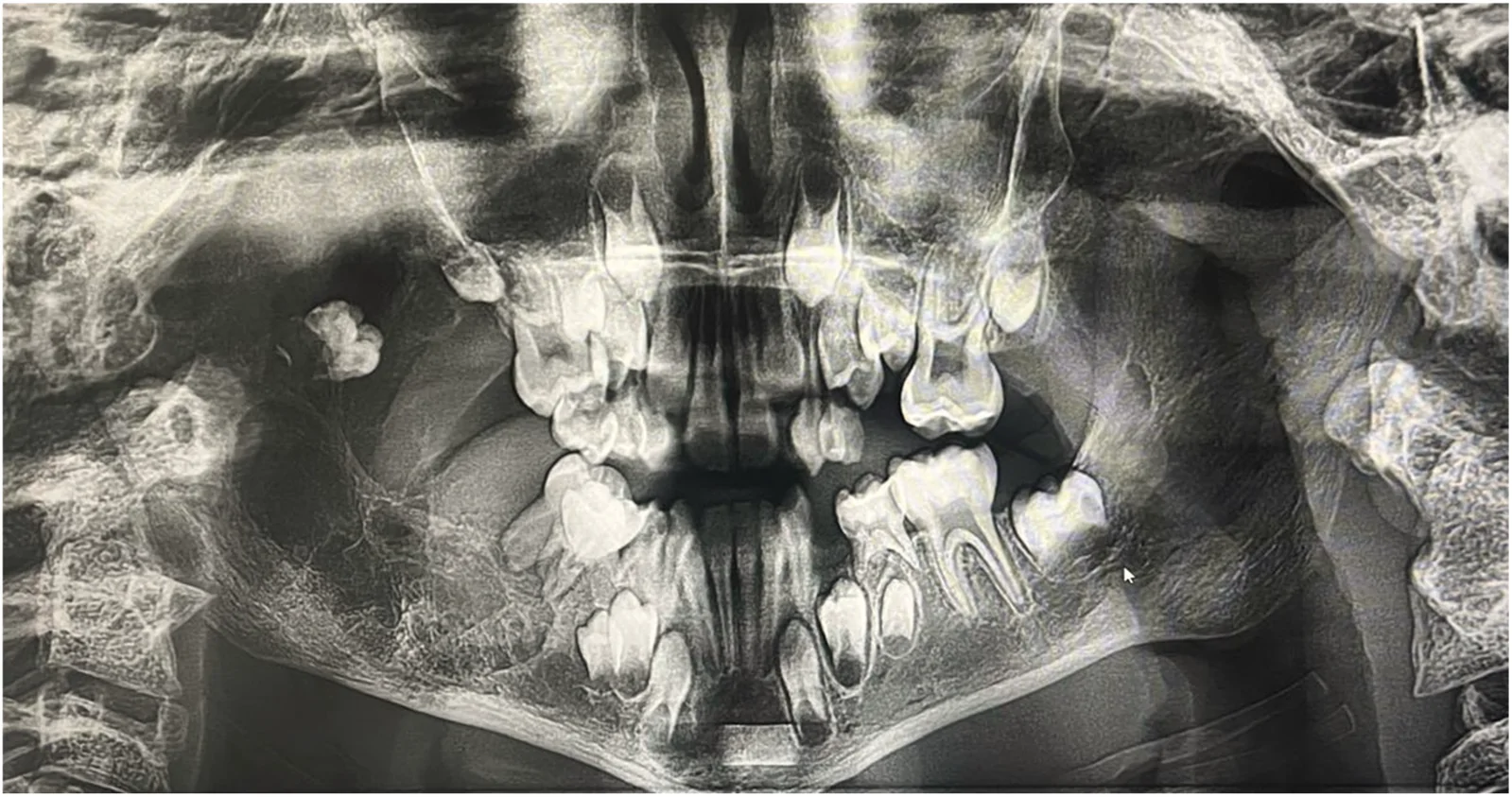
Panoramic radiograph showing: progression of the metastatic lesion in the region of the mesial root of tooth 46, with persistent arrest of root development. displacement of the tooth germs of teeth 44 and 45. Persistent displacement of tooth 47 toward the mandibular ramus, with ongoing coronal development.

**Figure 7:**
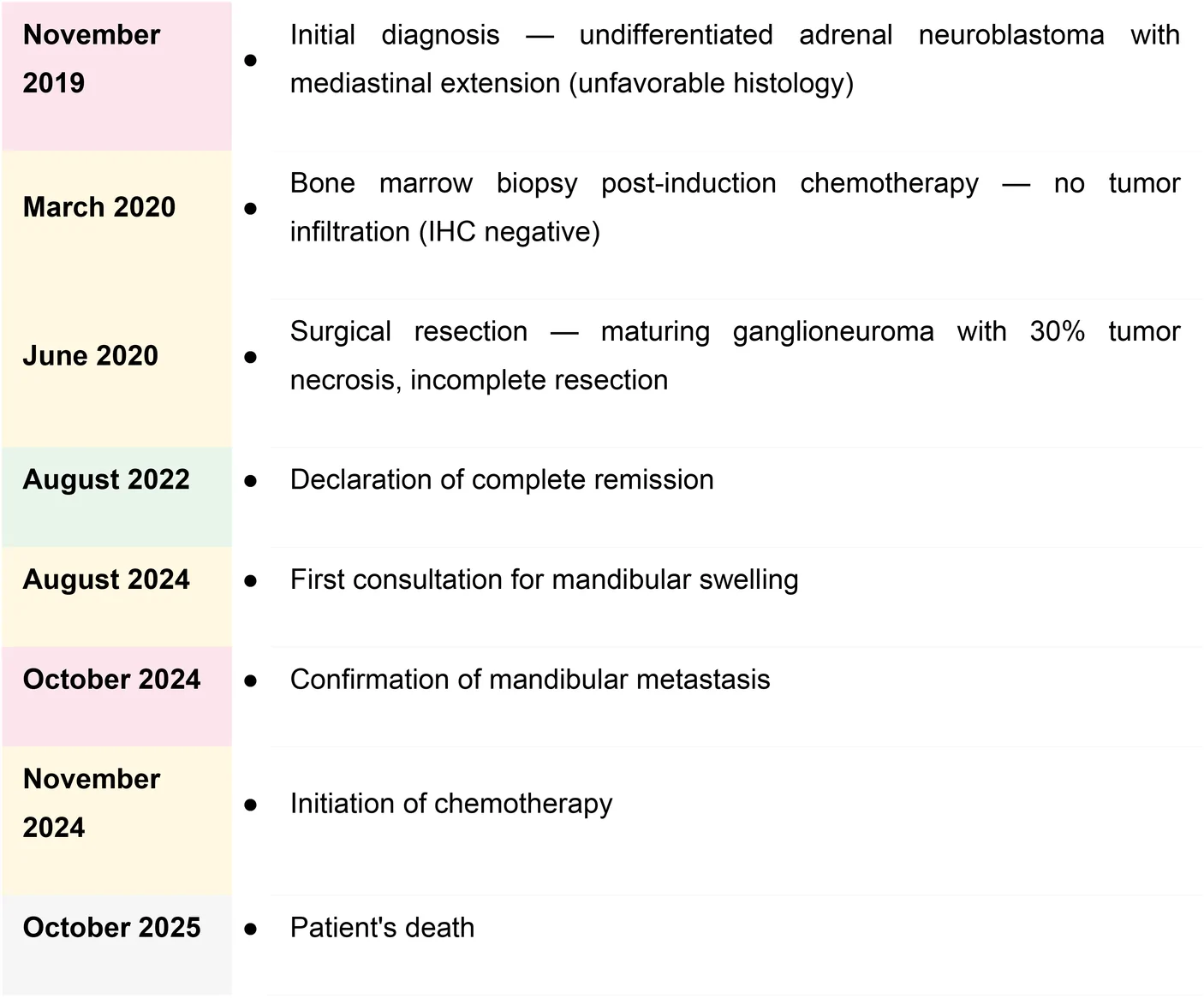
Clinical timeline of the patient's disease course.

At the 10-month post-treatment follow-up, intraoral and panoramic imaging demonstrated partial clinical regression of the soft-tissue lesion but continued radiographic progression of the mandibula (Figures 5 and 6). The sequence of clinical events, diagnostic investigations, treatment, and follow-up is summarized in Figure 7.

### Informed consent

Written informed consent for publication of the clinical information and photographic documentation was obtained from the patient's parents.

## Discussion

Neuroblastoma is the second most common solid malignancy in children after central nervous system tumors and represents the most common malignant tumor in infancy. Approximately 70% of cases are diagnosed before the age of 5 years, with nearly 30% occurring during the first year of life (9). In our case, the patient was diagnosed at the age of 4 years, which is consistent with these epidemiological findings. Although the etiology of neuroblastoma remains incompletely understood, advances in genomics and familial epidemiology have contributed to a better understanding of the genetic susceptibility factors involved in its development (10).

Neuroblastoma most commonly arises in the abdomen, particularly within the adrenal glands. The most frequent metastatic sites include the bone marrow, bones, lymph nodes, and liver, whereas intracranial, orbital, and head and neck metastases remain uncommon (11). Despite initial remission, relapse occurs in approximately 50%–60% of patients. Clinical manifestations vary depending on the location of the primary tumor and the extent of metastatic spread, and may be associated with nonspecific constitutional symptoms such as weight loss, fever, and asthenia. In the present case, the medical history revealed neuroblastoma in remission for two years, previously presenting as an aggressive undifferentiated tumor of the right adrenal gland with mediastinal extension. The primary tumor was of abdominal origin, in agreement with the data reported in the literature, and no systemic symptoms were observed. (9).

According to the literature published since 1952, most reported cases of mandibular metastases from neuroblastoma have been associated with cervicofacial manifestations such as tooth mobility, exophthalmos, and Horner's syndrome (11). In the present case, the patient presented with a painful mandibular swelling associated with tooth mobility.

Mandibular metastases of neuroblastoma remain exceptionally rare, with only a limited number of cases reported in the literature, estimated at approximately 17 to 20 cases depending on the published series over the last four decades. Owing to the rarity of this presentation, the available data remain insufficient to accurately determine its true frequency. This further highlights the exceptional nature of mandibular bone metastases, particularly when they constitute the inaugural manifestation of the disease (9, 11, 12).

Clinically, these lesions typically present as a rapidly enlarging mass associated with osteolytic bone destruction, tooth mobility or displacement, and alterations of the oral mucosa. The mandibular body and angle are reported to be the most frequently involved sites (4, 13).

The clinical findings observed in our patient were consistent with those reported in the literature, including lingual mobility of tooth 46, displacement of the tooth germ of 47 toward the ascending ramus, extensive osteolytic destruction of the alveolar ridge, and an erythematous lesion of the retromolar area mucosa.

The predilection of mandibular metastases for the molar region is thought to be related to its rich vascular supply and high concentration of hematopoietic marrow, which create a favorable environment for tumor cell implantation. Metastatic spread to the mandible occurs mainly through Batson's venous plexus and is facilitated by the presence of active hematopoietic bone marrow, which is closely associated with sinusoidal vascular spaces that promote tumor cell dissemination and seeding (11, 12, 14).

Because of its abundant vascularization, the mandibular body is the most frequently involved site, followed by the angle and the ascending ramus. Nevertheless, no region of the maxillomandibular skeleton is completely exempt from metastatic involvement, and in exceptional cases, the dental pulp may also be affected (4, 9, 12).

The radiographic features of bone metastases are variable and nonspecific, ranging from diffuse areas of osteolysis to extensive bone destruction with ill-defined borders, sometimes associated with a periosteal osteogenic reaction. In certain cases, perpendicular bone spicules extending into the adjacent soft tissues may be observed, producing a “sunburst” appearance similar to that classically described in osteosarcoma (4, 9, 15).

In our patient, the findings observed on panoramic radiography and cross-sectional imaging were consistent with these reported features, demonstrating significant cortical bone involvement. Based on these radiological characteristics, the main differential diagnoses considered were lymphoma, soft tissue sarcoma, and osteosarcoma. Most reported cases of mandibular metastases in the literature are symptomatic at presentation (16). The diagnosis is usually suspected on the basis of clinical examination and panoramic radiography, which may demonstrate nonspecific osteolytic lesions, and is subsequently confirmed by histopathological examination of a biopsy specimen obtained from the mucosal lesion (9). In our case, the possibility of recurrence was suspected early on based on the clinical examination and panoramic radiographic findings. Cross-sectional imaging was subsequently performed to assess the extent of the lesion. The diagnosis was ultimately confirmed by histopathological analysis of a biopsy specimen obtained from the mucosal lesion.

Metastatic neuroblastoma carries a variable prognosis depending on the patient's age and disease stage. Outcomes are generally more favorable in infants, whereas the disease tends to behave more aggressively and is associated with a poorer prognosis in older children. In children older than one year, the estimated 3-year event-free survival rate for metastatic neuroblastoma is approximately 15%. The presence of mandibular metastasis is considered a poor prognostic factor, usually reflecting advanced systemic dissemination of the disease (9, 17). Management is primarily palliative, relying on chemotherapy and/or radiotherapy depending on the characteristics of the primary tumor. Radical surgical resection may be considered in selected cases of solitary metastasis and may contribute to improved prognosis as well as better quality of life (12). In our patient, the extent of tumor involvement and the advanced stage of the disease led to the initiation of palliative chemotherapy.

At 10-month follow-up after treatment, intraoral examination showed a marked regression of the hyperplastic lesion compared with the initial presentation, accompanied by slight buccal repositioning of tooth 46 secondary to lesion shrinkage, although a persistent jugal swelling was still noted. The panoramic radiograph confirmed ongoing mandibular involvement, characterized by arrested root development of tooth 46, displacement of the tooth germs of teeth 44 and 45, and persistent inferior and posterior displacement of tooth 47 with continued coronal development.

Despite therapeutic advances, the prognosis of oral metastases remains poor, with more than 60% of patients dying within 12 months of diagnosis, largely due to the high incidence of synchronous metastatic disease at presentation (17). In our patient, the extent of tumor involvement and the advanced stage of the disease led to the initiation of palliative chemotherapy. The clinical course was unfavorable, characterized by the development of additional disseminated metastases, and the patient died 14 months after the diagnosis of mandibular metastasis.

This clinical case highlights the importance of a careful medical history and a thorough clinical examination for the early detection of secondary lesions. It also draws attention to the need for dental practitioners to remain highly vigilant when evaluating children with a history of malignancy or presenting with unexplained swelling or oral lesions. In such situations, early suspicion, prompt referral, and close collaboration with specialist teams are essential to ensure accurate diagnosis and optimal management. Early recognition of metastatic disease remains crucial for improving outcomes in children with neuroblastoma (15, 16).

## Data Availability

The original contributions presented in the study are included in the article/Supplementary Material, further inquiries can be directed to the corresponding author.
